# Sex‐Specific and Nonlinear Associations Between Serum 25‐Hydroxyvitamin D and Sex Hormones in US Adults

**DOI:** 10.1155/ije/8944835

**Published:** 2026-11-02

**Authors:** Yi Han, Simin Gao

**Affiliations:** ^1^ Anesthesia and Surgery Center of the Affiliated Hospital of North Sichuan Medical College Nanchong 637000 China; ^2^ Department of Otolaryngology-Head & Neck Surgery & Sleep Medicine Center West China School of Public Health and West China Fourth Hospital Sichuan University Chengdu 610041 China scu.edu.cn

**Keywords:** BMI, NHANES, nonlinear, sex hormones, SHBG, vitamin D

## Abstract

**Objective:**

This population‐based study sought to characterize sex‐specific linear and nonlinear associations between serum 25‐hydroxyvitamin D [25(OH)D] and four circulating sex hormone markers—total testosterone, estradiol, sex hormone–binding globulin (SHBG), and free testosterone index (FTI)—in the noninstitutionalized US adult population.

**Methods:**

We analyzed data from the 2021–2023 National Health and Nutrition Examination Survey (NHANES), including 4801 eligible participants aged 20 years and older (2169 men, 2632 women). A tiered analytical strategy was applied: weighted Spearman rank correlation for crude associations, multivariable weighted linear regression for adjusted effect estimates, restricted cubic spline (RCS) modeling for nonlinear dose–response characterization, and bootstrap resampling (500 replicates) for robustness validation.

**Results:**

In men, crude weak positive correlations of 25(OH)D with total testosterone (*r* = 0.07) and SHBG (*r* = 0.23) were fully attenuated to nonsignificance after adjustment for BMI and waist circumference; no robust nonlinear associations were identified via RCS. In women, 25(OH)D was moderately positively correlated with SHBG (*r* = 0.21), and this association remained significant after full adjustment (*β* = 0.148, *p* = 0.028), corresponding to a 10.8% increase in SHBG per doubling of 25(OH)D. RCS revealed a robust J‐shaped nonlinear relationship between 25(OH)D and SHBG (*p* for nonlinearity < 0.001), a decline‐then‐plateau pattern for FTI (*p* for nonlinearity = 0.015), an approximately linear negative trend for estradiol, and only a borderline nonlinear trend for total testosterone. Bootstrap analyses confirmed the robustness of SHBG, estradiol, and FTI associations in women.

**Conclusions:**

The association between vitamin D status and sex hormone profiles exhibits pronounced sex dimorphism. In women, SHBG most consistently correlates with 25(OH)D, showing a BMI‐independent positive association and a J‐shaped, nonlinear dose–response pattern. In men, adiposity indices (BMI and waist circumference) fully account for the crude associations of 25(OH)D with testosterone, estradiol, and SHBG.

## 1. Introduction

Beyond its well‐established function in calcium and phosphate homeostasis and bone maintenance, vitamin D—a lipid‐soluble steroid hormone—has been increasingly recognized for its extra‐skeletal actions. It is broadly involved in cardiovascular–kidney–metabolic (CKM) health, with the liver serving as a key organ in this interorgan crosstalk [1–3], insulin resistance, and the pathological processes of various endocrine and metabolic diseases. These include polyendocrine metabolic ovarian syndrome (PMOS), which affects multiple endocrine glands, and other endocrine and metabolic disorders [4–6]. Vitamin D deficiency is a widespread global issue and constitutes a significant public health concern. It is linked to an elevated risk of various chronic diseases.

The sex hormone system comprises total testosterone, estradiol, and SHBG, which jointly regulate reproductive development, energy metabolism, fat distribution, and insulin sensitivity. Disruption in their homeostasis is strongly associated with various diseases, including obesity, impaired CKM health, cardiovascular disease, and hypogonadism [7, 8]. Sex is an important regulatory factor in health, disease, and clinical medicine. There are significant differences in sex hormone profiles between men and women. Therefore, sex‐specific analysis is essential for understanding the mechanisms of disease and formulating individualized prevention and treatment strategies [9].

The vitamin D receptor (VDR) is present in multiple endocrine tissues, such as the testes, ovaries, uterus, liver, adipose tissue, and the hypothalamic–pituitary–gonadal axis [5, 10]. Animal and cellular studies suggest that vitamin D may influence sex hormone levels. This may occur through the regulation of steroidogenic enzyme expression and sex hormone synthesis pathways [11, 12]. These findings provide a potential biological basis for the association between vitamin D and sex hormones.

Nevertheless, findings from epidemiological studies remain conflicting. While some studies in men have reported a positive link between 25(OH)D and total testosterone, this relationship weakened or became nonsignificant after accounting for BMI [13, 14]. In women, the positive association between vitamin D and SHBG was relatively stable [15–17]; however, its relationship with estradiol and free testosterone remains controversial [16, 18, 19]. Notably, most studies have not fully explored nonlinear dose–response relationships. They have also not systematically assessed changes in these associations after adjustment for obesity‐related indices, such as BMI. Mendelian randomization evidence indicates that BMI may act as an upstream determinant of vitamin D insufficiency [20]. Consequently, BMI is associated with both vitamin D and sex hormones and may confound their relationship. Although BMI is easy to measure, reproducible, and cost‐effective for large epidemiological studies, it fails to differentiate fat from muscle and does not capture fat distribution, which are notable limitations [21]. In addition, the disease burden of BMI‐related metabolic disorders is increasing. These disorders include obesity and metabolic dysfunction–associated steatotic liver disease [21, 22]. Therefore, when examining the association between vitamin D and sex hormones, it is necessary to include BMI and waist circumference as covariates. It is also necessary to assess changes in the association before and after adjustment.

Building on these gaps in current evidence, this study evaluated sex‐specific associations between serum 25(OH)D and four sex hormone markers (total testosterone, estradiol, SHBG, and free testosterone index [FTI]) in the US adult population, with a specific focus on nonlinear dose–response patterns. We tested three a priori hypotheses: (1) associations between vitamin D and sex hormones differ by sex; (2) some associations are nonlinear; and (3) after adjustment for BMI and waist circumference, associations in men may be attenuated, whereas the SHBG association in women may be relatively independent.

## 2. Materials and Methods

### 2.1. Data Source

This study analyzed publicly available data from the 2021–2023 cycle of the National Health and Nutrition Examination Survey (NHANES), a nationally representative cross‐sectional survey administered by the US Centers for Disease Control and Prevention (CDC) National Center for Health Statistics (NCHS). NHANES employs a complex multistage stratified cluster probability sampling design to generate population‐representative estimates of health and nutritional status for the noninstitutionalized civilian US population. All participants provided written informed consent, and the survey protocol received full ethical approval from the NCHS Research Ethics Review Board. As the present study used only deidentified publicly accessible data, no additional institutional ethical review was required.

### 2.2. Research Object

We included civilian adults aged 20 years and older from the 2021–2023 NHANES cycle. Eligible participants were required to have completed both the household interview and mobile examination center (MEC) assessment, provided valid venous blood samples with complete measurements of serum 25(OH)D, total testosterone, estradiol, and SHBG, and had a valid venous blood draw sampling weight (WTPH2YR). Participants were excluded if they had missing data on educational attainment, family income‐to‐poverty ratio, any of the vitamin D or sex hormone markers, or sampling weights.

Of the 7809 adult participants aged ≥ 20 years in the 2021–2023 NHANES cycle, 4801 met all eligibility criteria and formed the final analytical sample (2169 men, 2632 women).

### 2.3. Variable Definition

#### 2.3.1. Exposure Variables

Vitamin D status was assessed by serum total 25(OH)D concentration (25OHD2 + 25OHD3, nmol/L). In the analysis, 25(OH)D was treated as both a continuous and categorical variable. According to the recommendations of the Endocrine Society, 25(OH)D was categorized into three groups: deficiency (< 50 nmol/L), insufficiency (50–74.9 nmol/L), and sufficiency (≥ 75 nmol/L) [23].

#### 2.3.2. Outcome Variables

Four sex hormone markers served as primary outcomes: total testosterone (nmol/L), estradiol (pmol/L), SHBG (nmol/L), and the FTI. FTI was calculated as the ratio of total testosterone to SHBG multiplied by 100, which is an indirect index for evaluating biologically active free testosterone levels in the body.

#### 2.3.3. Covariates

The demographic characteristics included age, sex, race (Mexican American, Other Hispanic, non‐Hispanic White, non‐Hispanic Black, and Other race), education level (less than high school, high school graduate, and college or above), and family income‐to‐poverty ratio.

Lifestyle factors included the following: (1) Smoking: this was defined as a binary variable based on SMQ020 (smoked ≥ 100 cigarettes in life) and SMQ040 (current smoking frequency). Categories were current smokers and noncurrent smokers. (2) Alcohol drinking: this was defined as a binary variable based on ALQ121 (frequency of alcohol drinking in the past 12 months). Categories were current drinkers and noncurrent drinkers.

Anthropometric measures included BMI and waist circumference.

### 2.4. Statistical Analysis

All statistical analyses were conducted in R Version 4.6.0. The “survey” package was used for complex sampling design adjustment, the “rms” package for restricted cubic spline (RCS) modeling, and the “ggplot2” package for data visualization. A two‐sided significance level of *α* = 0.05 was applied for all statistical tests.

#### 2.4.1. Complex Sampling Design

To generate population‐representative estimates for the US civilian noninstitutionalized adult population, all analyses incorporated the NHANES complex multistage sampling design. The WTPH2YR variable served as the sampling weight; SDMVSTRA (masked variance pseudo‐strata) and SDMVPSU (masked variance pseudo‐primary sampling units) were specified as stratification and primary sampling unit variables, respectively, to account for within‐cluster correlation. The survey design was defined using the “svydesign” function, with “singular.ok = TRUE” specified to accommodate strata containing only a single primary sampling unit.

#### 2.4.2. Descriptive Analysis

Weighted means (95% confidence intervals [CIs] for approximately normally distributed variables) or weighted medians (interquartile range [IQR] for skewed variables) were calculated for men and women stratified by the three categories of vitamin D status. Weighted percentages were calculated for the categorical variables. The skewed variables included 25(OH)D, total testosterone, estradiol, SHBG, and FTI. Between‐group comparisons were performed using weighted linear regression analysis for continuous variables. Tests were performed on log‐transformed values for skewed variables. Weighted logistic regression was used for the categorical variables.

#### 2.4.3. Weighted Correlation Analysis of Vitamin D and Sex Hormones

Sex hormone distributions were markedly right‐skewed. Therefore, weighted Spearman rank correlation was used to assess the unadjusted associations between continuous 25(OH)D and each sex hormone. These analyses were stratified by sex.

#### 2.4.4. Multivariate Weighted Linear Regression Analysis

Log‐transformed total testosterone, estradiol, SHBG, and FTI were used as the dependent variables. Log‐transformed 25(OH)D was used as the independent variable. Three progressively adjusted weighted linear regression models were built. Model 1 was adjusted for age and race. Model 2 was further adjusted for education, income, smoking, and alcohol consumption. Model 3 was further adjusted for BMI and waist circumference. All analyses were stratified by sex. Effect sizes were expressed as the percentage change per doubling of 25(OH)D.

#### 2.4.5. Nonlinear Relationship Test

RCS was used to assess potential nonlinear associations between log‐transformed 25(OH)D and log‐transformed sex hormones. Models were adjusted for age, race, education, income, smoking, alcohol consumption, BMI, and waist circumference. The “ols” function in the “rms” package was used to fit the models. Predicted curves and 95% CIs were plotted. To reduce the influence of extreme values, a sensitivity analysis was performed after excluding values outside the 1st and 99th percentiles of 25(OH)D.

#### 2.4.6. Robustness Analysis

To assess the impact of mild departures of the residuals from normality on the main results, we performed a bootstrap sensitivity analysis on the fully adjusted model. This analysis used 500 replicates. Bootstrap standard errors and 95% CIs were calculated. This analysis did not incorporate the complex sampling design. It served only as supportive evidence.

## 3. Results

### 3.1. Baseline Characteristics of the Study Population

This study included 4801 US adults aged ≥ 20 years from the NHANES 2021–2023 cycle (2169 men and 2632 women). After applying complex sampling weights, these participants represented approximately 202 million US adults. Because sex hormone profiles differ significantly by sex, baseline characteristics were presented separately by sex (Tables 1 and 2).

**TABLE 1:** Weighted baseline characteristics of male participants by vitamin D status.

Variable	Deficient	Insufficient	Sufficient	*p*
Weighted *N*	21,050,499	34,103,162	43,357,529	
Age (years)	42.76 (40.05, 45.46)	45.11 (43.66, 46.55)	53.77 (51.58, 55.97)	< 0.001
BMI (kg/m^2^)	30.57 (29.61, 31.54)	29.57 (28.90, 30.24)	28.70 (28.09, 29.31)	0.002
Waist circumference (cm)	104.79 (102.11, 107.46)	103.29 (101.61, 104.96)	102.33 (100.68, 103.98)	0.127
Vitamin D (nmol/L)	37.50 (30.10, 45.10)	63.00 (56.80, 69.10)	93.00 (83.10, 110.00)	< 0.001
Total testosterone (nmol/L)	13.60 (9.92, 18.80)	15.10 (10.60, 19.70)	15.20 (11.50, 19.70)	0.055
Estradiol (pmol/L)	90.30 (66.80, 110.00)	85.50 (66.40, 107.00)	85.10 (62.00, 111.00)	0.626
SHBG (nmol/L)	27.69 (20.33, 38.99)	32.46 (22.52, 42.92)	37.46 (26.35, 51.35)	< 0.001
FTI	48.52 (36.33, 65.32)	46.81 (34.62, 61.50)	40.72 (30.79, 53.59)	< 0.001
Race—Mexican American	11.97 (2.24, 21.70)	8.91 (3.97, 13.85)	4.72 (2.09, 7.36)	0.018
Race—other Hispanic	13.20 (8.67, 17.73)	8.52 (4.75, 12.29)	6.90 (3.10, 10.69)	0.103
Race—non‐Hispanic White	39.65 (33.28, 46.01)	63.31 (55.58, 71.04)	72.78 (69.12, 76.45)	< 0.001
Race—non‐Hispanic Black	20.11 (12.54, 27.69)	7.45 (5.71, 9.19)	4.97 (3.73, 6.21)	< 0.001
Race—other race	15.07 (11.39, 18.76)	11.81 (8.66, 14.96)	10.63 (6.86, 14.39)	0.164
Education level—less than high school	14.89 (10.28, 19.50)	11.32 (7.20, 15.43)	7.38 (5.41, 9.36)	0.003
Education level—high school	27.09 (20.59, 33.59)	27.10 (21.27, 32.93)	26.30 (21.77, 30.82)	0.942
Education level—college or above	58.02 (51.23, 64.80)	61.58 (53.93, 69.23)	66.32 (60.57, 72.06)	0.021
Smoking status—current	25.48 (21.26, 29.71)	17.70 (14.05, 21.35)	14.10 (10.58, 17.62)	< 0.001
Smoking status—noncurrent	74.52 (70.29, 78.74)	82.30 (78.65, 85.95)	85.90 (82.38, 89.42)	< 0.001
Alcohol use—current	72.95 (66.21, 79.69)	68.97 (64.99, 72.94)	72.68 (68.29, 77.06)	0.512
Alcohol use—noncurrent	27.05 (20.31, 33.79)	31.03 (27.06, 35.01)	27.32 (22.94, 31.71)	0.512

*Note:* Values are presented as weighted means with 95% confidence intervals for approximately normally distributed variables, weighted medians with interquartile ranges (Q1, Q3) for skewed variables, and weighted percentages with 95% confidence intervals for categorical variables.

Abbreviations: BMI, body mass index; FTI, free testosterone index; SHBG, sex hormone–binding globulin.

**TABLE 2:** Weighted baseline characteristics of female participants by vitamin D status.

Variable	Deficient	Insufficient	Sufficient	*p*
Weighted *N*	19,252,993	26,580,133	57,727,264	
Age (years)	40.43 (38.89, 41.97)	43.65 (42.13, 45.18)	54.44 (52.23, 56.65)	< 0.001
BMI (kg/m^2^)	32.24 (30.78, 33.70)	30.92 (30.02, 31.82)	28.96 (28.50, 29.43)	< 0.001
Waist circumference (cm)	101.49 (98.23, 104.75)	99.20 (96.97, 101.43)	96.45 (95.35, 97.55)	0.009
Vitamin D (nmol/L)	36.90 (29.00, 43.30)	63.20 (56.80, 69.10)	101.00 (86.70, 123.00)	< 0.001
Total testosterone (nmol/L)	0.87 (0.58, 1.35)	0.81 (0.53, 1.18)	0.72 (0.46, 1.04)	< 0.001
Estradiol (pmol/L)	170.00 (49.90, 418.00)	139.00 (26.10, 382.00)	30.20 (11.50, 193.00)	< 0.001
SHBG (nmol/L)	43.40 (30.16, 67.60)	47.11 (32.64, 69.02)	59.21 (39.74, 87.04)	< 0.001
FTI	1.93 (1.17, 2.97)	1.65 (1.07, 2.70)	1.20 (0.67, 1.96)	< 0.001
Race—Mexican American	12.37 (4.47, 20.28)	7.62 (2.79, 12.45)	3.15 (1.85, 4.45)	< 0.001
Race—other Hispanic	13.41 (8.04, 18.79)	14.42 (10.02, 18.83)	5.64 (3.42, 7.86)	< 0.001
Race—non‐Hispanic White	29.72 (25.08, 34.36)	56.87 (51.00, 62.74)	74.10 (70.83, 77.36)	< 0.001
Race—non‐Hispanic Black	25.60 (20.05, 31.16)	10.85 (7.21, 14.49)	6.90 (4.48, 9.33)	< 0.001
Race—other race	18.89 (11.13, 26.64)	10.24 (6.45, 14.03)	10.21 (7.77, 12.64)	0.040
Education level—less than high school	13.28 (9.04, 17.51)	9.85 (6.49, 13.21)	5.77 (4.16, 7.37)	< 0.001
Education level—high school	32.98 (26.06, 39.90)	24.53 (19.16, 29.89)	21.96 (18.41, 25.50)	0.013
Education level—college or above	53.74 (46.30, 61.19)	65.62 (59.55, 71.69)	72.28 (67.55, 77.01)	< 0.001
Smoking status—current	14.35 (10.21, 18.50)	11.01 (7.66, 14.36)	10.99 (8.78, 13.21)	0.198
Smoking status—noncurrent	85.65 (81.50, 89.79)	88.99 (85.64, 92.34)	89.01 (86.79, 91.22)	0.198
Alcohol use—current	63.89 (57.29, 70.49)	62.44 (57.70, 67.18)	70.52 (68.42, 72.61)	0.023
Alcohol use—noncurrent	36.11 (29.51, 42.71)	37.56 (32.82, 42.30)	29.48 (27.39, 31.58)	0.023

*Note:* Values are presented as weighted means with 95% confidence intervals for approximately normally distributed variables, weighted medians with interquartile ranges (Q1, Q3) for skewed variables, and weighted percentages with 95% confidence intervals for categorical variables.

Abbreviations: BMI, body mass index; FTI, free testosterone index; SHBG, sex hormone–binding globulin.

Among men, the weighted population proportions of vitamin D deficiency, insufficiency, and sufficiency were 21.37%, 34.62%, and 44.01%, respectively (Table 1). Participants in the sufficiency group were significantly older (53.77 vs. 42.76 years, *p* < 0.001) and had lower BMI (28.70 vs. 30.57 kg/m^2^, *p* = 0.002) than those in the deficiency group. Waist circumference did not differ statistically across the three categories (104.79, 103.29, 102.33 cm; *p* = 0.127). For sex hormone markers, median total testosterone exhibited a borderline increasing gradient across vitamin D categories (13.60, 15.10, 15.20 nmol/L; *p* = 0.055), while SHBG increased monotonically and significantly (27.69, 32.46, 37.46 nmol/L; *p* < 0.001). FTI declined significantly with improving vitamin D status (48.52, 46.81, 40.72; *p* < 0.001), whereas no significant between‐group difference was observed for estradiol (90.30, 85.50, 85.10 pmol/L; *p* = 0.626). The current smoking rate was highest in the deficiency group (25.48% vs. 14.10%, *p* < 0.001). Drinking status did not differ significantly among the three groups (*p* = 0.512).

In women, the weighted proportions of the vitamin D deficiency, insufficiency, and sufficiency groups were 18.59%, 25.67%, and 55.74%, respectively (Table 2). The vitamin D sufficiency group had the highest age (54.44 vs. 40.43 years, *p* < 0.001), the lowest BMI (28.96 vs. 32.24 kg/m^2^, *p* < 0.001), and the lowest waist circumference (96.45 vs. 101.49 cm, *p* = 0.009). Total testosterone decreased significantly with higher vitamin D levels (medians: 0.87, 0.81, and 0.72 nmol/L, *p* < 0.001). SHBG increased significantly with higher vitamin D levels (medians: 43.40, 47.11, and 59.21 nmol/L, *p* < 0.001). FTI decreased significantly (medians: 1.93, 1.65, and 1.20, *p* < 0.001). Estradiol showed a significant downward trend (medians: 170.00, 139.00, and 30.20 pmol/L, *p* < 0.001). However, its IQR was extremely wide. This was especially true in the sufficiency group, with a median (IQR) of 30.20 (11.50, 193.00) pmol/L. Therefore, this result should be interpreted with caution. The current smoking rate did not differ significantly among the three groups (*p* = 0.198). Drinking status differed significantly (*p* = 0.023).

### 3.2. Weighted Correlation Analysis of Vitamin D and Sex Hormones

Sex hormone indicators showed a markedly right‐skewed distribution. Therefore, unadjusted correlation analysis was performed using weighted Spearman rank correlation (Table 3). In men, 25(OH)D was weakly positively correlated with total testosterone (*r* = 0.07, *p* = 0.002). It was moderately positively correlated with SHBG (*r* = 0.23, *p* < 0.001). It was weakly negatively correlated with FTI (*r* = −0.19, *p* < 0.001). The correlation with estradiol was very weak and marginally significant (*r* = −0.04, *p* = 0.039). In women, 25(OH)D was negatively correlated with total testosterone (*r* = −0.15, *p* < 0.001), estradiol (*r* = −0.32, *p* < 0.001), and FTI (*r* = −0.27, *p* < 0.001). It was moderately positively correlated with SHBG (*r* = 0.21, *p* < 0.001). All correlations were unadjusted. They did not account for confounders, such as age, race, socioeconomic status, lifestyle, BMI, and waist circumference.

**TABLE 3:** Weighted Spearman correlations between 25(OH)D and sex hormones by sex.

Sex	Hormone	Spearman_*r*	*p*
Male	Total testosterone	0.07	0.002
Male	Estradiol	−0.04	0.039
Male	SHBG	0.23	< 0.001
Male	FTI	−0.19	< 0.001
Female	Total testosterone	−0.15	< 0.001
Female	Estradiol	−0.32	< 0.001
Female	SHBG	0.21	< 0.001
Female	FTI	−0.27	< 0.001

### 3.3. Multivariate Weighted Linear Regression Analysis

Three progressively adjusted weighted linear regression models were used. The exposure variable was log‐transformed 25(OH)D. The outcome variables were log‐transformed sex hormone measures. Model 1 was adjusted for age and race. Model 2 was further adjusted for education, income, smoking, and alcohol consumption. Model 3 was further adjusted for BMI and waist circumference (Table 4). Effect sizes were expressed as the percentage change per doubling of 25(OH)D.

**TABLE 4:** Multivariable weighted linear regression of log‐transformed 25(OH)D and log‐transformed sex hormones by sex.

Sex	Hormone	Model	*β* (95% CI)	*p*	Percent_change_per_doubling
Male	Total testosterone	Model 1	0.114 (0.055, 0.173)	0.002	8.2
Male	Total testosterone	Model 2	0.115 (0.040, 0.191)	0.013	8.3
Male	Total testosterone	Model 3	0.052 (−0.082, 0.185)	0.237	3.7
Male	Estradiol	Model 1	−0.066 (−0.115, −0.017)	0.014	−4.5
Male	Estradiol	Model 2	−0.077 (−0.139, −0.014)	0.027	−5.2
Male	Estradiol	Model 3	−0.043 (−0.142, 0.055)	0.200	−3.0
Male	SHBG	Model 1	0.070 (0.012, 0.128)	0.024	5.0
Male	SHBG	Model 2	0.084 (0.008, 0.159)	0.037	6.0
Male	SHBG	Model 3	0.033 (−0.068, 0.135)	0.293	2.3
Male	Free testosterone index	Model 1	0.044 (−0.025, 0.113)	0.182	3.1
Male	Free testosterone index	Model 2	0.031 (−0.052, 0.115)	0.354	2.2
Male	Free testosterone index	Model 3	0.018 (−0.117, 0.154)	0.617	1.3
Female	Total testosterone	Model 1	−0.031 (−0.106, 0.045)	0.383	−2.1
Female	Total testosterone	Model 2	−0.038 (−0.139, 0.062)	0.349	−2.6
Female	Total testosterone	Model 3	−0.036 (−0.205, 0.133)	0.457	−2.5
Female	Estradiol	Model 1	−0.203 (−0.304, −0.101)	0.001	−13.1
Female	Estradiol	Model 2	−0.224 (−0.357, −0.091)	0.009	−14.4
Female	Estradiol	Model 3	−0.210 (−0.454, 0.034)	0.066	−13.5
Female	SHBG	Model 1	0.231 (0.171, 0.291)	< 0.001	17.3
Female	SHBG	Model 2	0.236 (0.158, 0.315)	0.001	17.8
Female	SHBG	Model 3	0.148 (0.039, 0.258)	0.028	10.8
Female	Free testosterone index	Model 1	−0.262 (−0.342, −0.181)	< 0.001	−16.6
Female	Free testosterone index	Model 2	−0.275 (−0.380, −0.170)	0.002	−17.3
Female	Free testosterone index	Model 3	−0.184 (−0.376, 0.008)	0.054	−12.0

*Note:* Model 1 was adjusted for age and race; Model 2 was further adjusted for education, income, smoking, and alcohol use; Model 3 was additionally adjusted for BMI and waist circumference.

Abbreviations: FTI, free testosterone index; SHBG, sex hormone–binding globulin.

In men, 25(OH)D was positively associated with total testosterone in Model 1 and Model 2. Model 1: *β* = 0.114, 95% CI 0.055–0.173, *p* = 0.002, corresponding to an 8.2% increase per doubling of 25(OH)D. Model 2: *β* = 0.115, 95% CI 0.040–0.191, *p* = 0.013, corresponding to an 8.3% increase. However, after additional adjustment for BMI and waist circumference in Model 3, this association was no longer significant. Model 3: *β* = 0.052, 95% CI −0.082–0.185, *p* = 0.237, corresponding to a 3.7% increase. SHBG showed a similar pattern. It was significant in Model 1 (*β* = 0.070, 95% CI 0.012–0.128, *p* = 0.024, corresponding to a 5.0% increase) and Model 2 (*β* = 0.084, 95% CI 0.008–0.159, *p* = 0.037, corresponding to a 6.0% increase). It was attenuated in Model 3 (*β* = 0.033, 95% CI −0.068–0.135, *p* = 0.293, corresponding to a 2.3% increase). Estradiol was negatively associated in Model 1 and Model 2. Model 1: *β* = −0.066, 95% CI −0.115–−0.017, *p* = 0.014, corresponding to a 4.5% decrease. Model 2: *β* = −0.077, 95% CI −0.139–−0.014, *p* = 0.027, corresponding to a 5.2% decrease. It was not significant in Model 3 (*β* = −0.043, 95% CI −0.142–0.055, *p* = 0.200, corresponding to a 3.0% decrease). FTI was not significant in any model (all *p* > 0.05).

In women, total testosterone was not significantly associated in any model (all *p* > 0.05). SHBG was the only marker that remained significantly positively associated in Model 3. Model 1: *β* = 0.231, 95% CI 0.171–0.291, *p* < 0.001, corresponding to a 17.3% increase. Model 2: *β* = 0.236, 95% CI 0.158–0.315, *p* = 0.001, corresponding to a 17.8% increase. Model 3: *β* = 0.148, 95% CI 0.039–0.258, *p* = 0.028, corresponding to a 10.8% increase. Estradiol was negatively associated in Models 1 and 2. Model 1: *β* = −0.203, 95% CI −0.304 to −0.101, *p* = 0.001, corresponding to a 13.1% decrease. Model 2: *β* = −0.224, 95% CI −0.357 to −0.091, *p* = 0.009, corresponding to a 14.4% decrease. In Model 3, this association was of borderline significance (*β* = −0.210, 95% CI −0.454 to 0.034, *p* = 0.066, corresponding to a 13.5% decrease). FTI was also significantly negatively associated in Models 1 and 2. Model 1: *β* = −0.262, 95% CI −0.342 to −0.181, *p* < 0.001, corresponding to a 16.6% decrease. Model 2: *β* = −0.275, 95% CI −0.380 to −0.170, *p* = 0.002, corresponding to a 17.3% decrease. In Model 3, this association was of borderline significance (*β* = −0.184, 95% CI −0.376 to 0.008, *p* = 0.054, corresponding to a 12.0% decrease).

These findings suggest that BMI and waist circumference are important confounders of the associations of 25(OH)D with total testosterone, estradiol, and SHBG in men. In women, SHBG was the most stable target of 25(OH)D. Its association was independent of BMI and waist circumference. Estradiol and FTI may still be negatively associated with 25(OH)D independently of BMI in women.

### 3.4. Nonlinear Correlation Analysis Between Vitamin D and Sex Hormones

RCS was used to evaluate nonlinear dose–response relationships between 25(OH)D and sex hormones. Models were adjusted for age, race, education, income, smoking, alcohol consumption, BMI, and waist circumference. To minimize the influence of extreme values, a sensitivity analysis was also performed after excluding values below the 1st percentile or above the 99th percentile of log‐transformed 25(OH)D (Table 5).

**TABLE 5:** RCS nonlinearity tests for 25(OH)D and sex hormones by sex.

Sex	Hormone	Analysis	Nonlin_*p*
Male	Total testosterone	Main	0.760
Male	Estradiol	Main	0.561
Male	SHBG	Main	0.056
Male	Free testosterone index	Main	0.414
Female	Total testosterone	Main	0.050
Female	Estradiol	Main	0.705
Female	SHBG	Main	< 0.001
Female	Free testosterone index	Main	0.015
Male	Total testosterone	Sensitivity (exclude 1% extremes)	0.581
Male	Estradiol	Sensitivity (exclude 1% extremes)	0.995
Male	SHBG	Sensitivity (exclude 1% extremes)	0.580
Male	Free testosterone index	Sensitivity (exclude 1% extremes)	0.528
Female	Total testosterone	Sensitivity (exclude 1% extremes)	0.067
Female	Estradiol	Sensitivity (exclude 1% extremes)	0.957
Female	SHBG	Sensitivity (exclude 1% extremes)	< 0.001
Female	Free testosterone index	Sensitivity (exclude 1% extremes)	0.036

In men, no consistent nonlinear associations were observed (Figure 1). In the main analysis, the *p* values for nonlinearity were 0.760 for total testosterone, 0.561 for estradiol, 0.056 for SHBG, and 0.414 for FTI. In the sensitivity analysis, all *p* values for nonlinearity were > 0.05 (0.581 for total testosterone, 0.995 for estradiol, 0.580 for SHBG, and 0.528 for FTI). SHBG showed borderline nonlinearity in the main analysis. However, this nonlinearity disappeared in the sensitivity analysis. This finding suggests that the nonlinear association was not robust.

**FIGURE 1:**
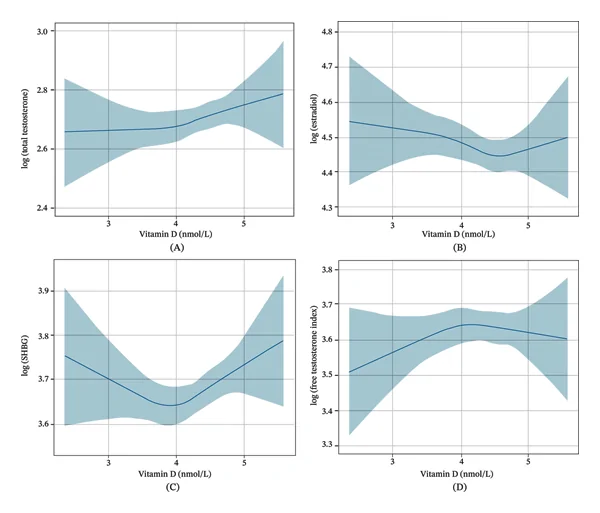
Nonlinear relationships between vitamin D and sex hormones among men.

In women, the nonlinear associations of SHBG and FTI were robust (Figure 2). SHBG showed a classic J‐shaped nonlinear pattern. The *p* values for nonlinearity were < 0.001 in both the main and sensitivity analyses. The nadir occurred at approximately 50–60 nmol/L. Thereafter, SHBG increased with higher 25(OH)D levels. FTI showed a nonlinear negative association. The *p* value was 0.015 in the main analysis and 0.036 in the sensitivity analysis. The pattern was a decline followed by a leveling‐off. The inflection point occurred at approximately 100–120 nmol/L. Total testosterone showed a borderline nonlinear trend. The *p* value was 0.050 in the main analysis and 0.067 in the sensitivity analysis. However, the CIs were wide. Therefore, this finding should be interpreted with caution. Estradiol showed an approximately linear negative association, with no evidence of nonlinearity. The *p* value was 0.705 in the main analysis and 0.957 in the sensitivity analysis. In addition, the sensitivity analysis plots after excluding extreme values for men and women are provided in Supporting Figures S1 and S2, respectively. The corresponding log‐scale scatter plots for men and women are provided in Supporting Figure S3 and Supporting Figure S4, respectively.

**FIGURE 2:**
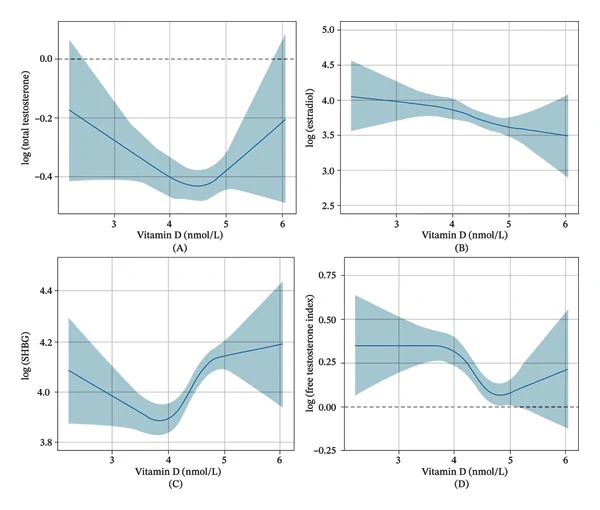
Nonlinear relationships between vitamin D and sex hormones among women.

### 3.5. Robustness Analysis

To assess the impact of mild departures from normality in the residuals, a bootstrap sensitivity analysis (500 replicates) was performed on the fully adjusted model (Model 3) (Table 6). In men, all associations remained nonsignificant in the bootstrap analysis (total testosterone: *β* = 0.052, 95% CI −0.006 to 0.107, *p* = 0.090; estradiol: *β* = −0.043, 95% CI −0.104 to 0.010, *p* = 0.135; SHBG: *β* = 0.033, 95% CI −0.019 to 0.085, *p* = 0.228; and FTI: *β* = 0.019, 95% CI −0.044 to 0.080, *p* = 0.550). In women, the positive association with SHBG was the most robust (*β* = 0.148, 95% CI 0.083–0.220, *p* < 0.001). The negative associations of estradiol (*β* = −0.210, 95% CI −0.368 to −0.060, *p* = 0.011) and FTI (*β* = −0.184, 95% CI −0.273 to −0.107, *p* < 0.001) were also robust in the bootstrap analysis. However, these associations were only borderline significant in the main analysis. Total testosterone was not significant in any bootstrap model (*β* = −0.036, 95% CI −0.113 to 0.040, *p* = 0.340). These results were consistent in direction with the main analysis and supported the robustness of the primary findings. The bootstrap analysis did not incorporate the complex sampling design. It served only as supportive evidence.

**TABLE 6:** Bootstrap sensitivity analysis for fully adjusted models (500 replicates).

Sex	Hormone	*β*	SE	95% CI	*p*	Percent_change_per_doubling
Male	Total testosterone	0.052	0.031	−0.006, 0.107	0.090	3.7
Male	Estradiol	−0.043	0.029	−0.104, 0.010	0.135	−3.0
Male	SHBG	0.033	0.028	−0.019, 0.085	0.228	2.3
Male	Free testosterone index	0.019	0.031	−0.044, 0.080	0.550	1.3
Female	Total testosterone	−0.036	0.038	−0.113, 0.040	0.340	−2.5
Female	Estradiol	−0.210	0.082	−0.368, 0.060	0.011	−13.5
Female	SHBG	0.148	0.035	0.083, 0.220	< 0.001	10.8
Female	Free testosterone index	−0.184	0.043	−0.273, −0.107	< 0.001	−12.0

## 4. Discussion

This study used nationally representative data from the 2021–2023 NHANES. We evaluated sex‐specific associations between serum 25(OH)D and total testosterone, estradiol, SHBG, and FTI. Models were adjusted for covariates including BMI and waist circumference. The main findings were as follows. (1) In women, SHBG was the most consistent correlate of 25(OH)D. It remained significantly positively associated after adjustment for BMI and waist circumference. It also showed a J‐shaped nonlinear pattern. (2) In men, the associations of 25(OH)D with total testosterone, estradiol, and SHBG disappeared or were attenuated after adjustment for BMI and waist circumference. No robust nonlinear associations were observed. (3) In women, FTI showed a decline‐then‐plateau nonlinear pattern. Estradiol showed an approximately linear negative association. Total testosterone showed only a borderline nonlinear trend. These findings provide epidemiological evidence to improve understanding of the sex‐specific associations between vitamin D and sex hormones.

In our analysis, the crude positive association between serum 25(OH)D and total testosterone in men was fully attenuated and lost statistical significance after additional adjustment for BMI and waist circumference. This pattern converges with findings across multiple ethnic and age groups. Chin et al. [13] documented a comparable loss of significance following BMI adjustment in a Malaysian adult male cohort. Damas‐Fuentes et al. [14] also reported that in obese young men, the association between 25(OH)D and total testosterone disappeared after adjustment for BMI, although a direct effect persisted only in the morbidly obese subgroup. Consistent results were also reported in older Chinese adults, in whom the vitamin D–testosterone correlation was abolished after BMI control, and body fat percentage was inversely associated with vitamin D status [24]. Collectively, this cross‐population evidence suggests that BMI may act as a confounder and potential effect modifier of the vitamin D–testosterone relationship in men. Vimaleswaran et al. [20] used bidirectional Mendelian randomization analysis to demonstrate that high BMI leads to low 25(OH)D. In contrast, low 25(OH)D does not lead to increased BMI. Chen et al. [25] further confirmed a causal relationship between genetically determined low 25(OH)D levels and low total testosterone levels using Mendelian randomization. These genetic findings suggest that BMI may be indirectly associated with sex hormones by affecting vitamin D levels. However, this pathway could not be verified in the present study. Therefore, no causal interpretation was made regarding the role of BMI.

At the mechanistic level, Hofer et al. [11] demonstrated in human primary testicular cells that 1,25‐dihydroxyvitamin D3 [1,25(OH)2D3] directly upregulated the expression of steroidogenic enzymes, including CYP11A1 and HSD3B2. It also significantly increased testosterone synthesis. Wang et al. [12] further found, using a VDR‐knockout mouse model, that VDR deletion led to abnormal testicular lipid metabolism, downregulation of Cyp11a1 and Hsd3b1 expression, and reduced testosterone synthesis. However, this effect may only be fully realized when vitamin D levels are adequate and the individual is not obese. Obesity causes fat tissue to store large amounts of vitamin D, leading to a reduction in the amount of active vitamin D in the bloodstream; at the same time, obesity can also cause persistent low‐grade inflammation and insulin resistance. Together, these factors may weaken the beneficial effects that vitamin D would otherwise have on testosterone.

Evidence from randomized controlled trials (RCTs) stands in contrast to the positive associations consistently observed in observational studies. Two RCTs by Lerchbaum and colleagues—one in healthy men [26] and another in men with low testosterone [27]—uniformly demonstrated no significant changes in total testosterone, free testosterone, or SHBG concentrations. Saha et al. [28] similarly reported no testosterone elevation following combined vitamin D and calcium supplementation in young Indian adult males. A 2022 meta‐analysis of 10 RCTs found only a marginally significant effect of vitamin D supplementation on total testosterone (SMD = 0.133, 95% CI: −0.003 to 0.269, *p* = 0.056) [29]. Collectively, the RCT evidence base supports the interpretation that the positive vitamin D–testosterone correlation seen in observational settings is largely driven by confounding factors—particularly BMI—rather than reflecting a direct causal relationship.

In women, we identified a J‐shaped positive dose–response association between 25(OH)D and SHBG, with an inflection point at approximately 50–60 nmol/L. This association remained statistically significant after full adjustment for BMI and waist circumference (adjusted *β* = 0.148, *p* = 0.028), corresponding to a 10.8% relative increase in SHBG per doubling of 25(OH)D. These findings align with prior population‐based evidence: Chu et al. [16] reported a similar positive correlation (*r* = 0.195, *p* < 0.001) in reproductive‐aged women; Balogh et al. [15] documented a stronger association (*r* = 0.29, *p* < 0.01) in women with PMOS, and Zhao et al. [17] confirmed a sex‐stratified effect with stronger associations in women in the multiethnic MESA cohort. At the molecular level, a comprehensive review by Xing et al. [8] delineated the transcriptional mechanism underlying this association: vitamin D signaling upregulates hepatic SHBG synthesis via activation of the hepatocyte nuclear factor 4 alpha (HNF‐4α) pathway.

The J‐shaped pattern observed in women may be explained by the graded effects of vitamin D deficiency severity on endocrine function. In primary human ovarian theca cells, severe 1,25(OH)_2_D_3_ deficiency (< 2 nM) has been shown to suppress androstenedione biosynthesis and downregulate insulin receptor expression, whereas mild vitamin D insufficiency exerts only marginal inhibitory effects [30]. Building on this experimental evidence, we propose a two‐phase mechanistic hypothesis to account for the observed J‐curve. Below the 50 nmol/L threshold, low circulating 25(OH)D may indirectly suppress hepatic SHBG production via secondary hyperparathyroidism or systemic insulin resistance. Once vitamin D status surpasses this cutoff, VDR‐mediated direct transcriptional upregulation of SHBG becomes the predominant driving force, underlying the steep increase in circulating SHBG concentrations with higher 25(OH)D levels.

Unlike in women, the positive correlation between SHBG and vitamin D in men in this study disappeared after adjusting for BMI. This gender difference may be related to the sex hormone environment, as estrogen upregulates SHBG gene expression, whereas androgens have the opposite effect. Higher estrogen levels in women may synergize with vitamin D to enhance SHBG transcription, rendering the effects of vitamin D independent of obesity, whereas in men, obesity‐related insulin resistance may attenuate the direct stimulatory effect of vitamin D on SHBG by reducing VDR sensitivity in hepatocytes [8, 17].

In this study, 25(OH)D was inversely associated with estradiol and FTI in women. After adjustment for BMI, the *p* values were 0.066 and 0.054, respectively, indicating borderline significance. Bootstrap sensitivity analysis supported the robustness of these associations. Knight et al. [31] found that in young healthy women, each 10 nmol/L increase in 25(OH)D was associated with approximately 3% lower luteal‐phase estradiol and approximately 10% lower progesterone. Chu et al. [16] also reported a negative association between 25(OH)D and FTI (*r* = −0.229, *p* < 0.001). Mechanistically, Seaward Brain et al. [30] demonstrated that severe vitamin D deficiency can inhibit cAMP‐driven estradiol synthesis; furthermore, vitamin D may reduce the conversion of androstenedione to estradiol by inhibiting aromatase activity in adipose tissue. It is worth noting that in this study, the RCS curve for female FTI showed a “decreasing then flattening” trend, with an inflection point at approximately 100–120 nmol/L. This pattern suggests that the association between vitamin D and free androgens tends to level off at higher levels; however, this study cannot determine whether this represents an “optimal range.” A systematic review by Jaafar et al. [4] noted that the effects of vitamin D supplementation on androgen levels in women with PMOS were inconsistent, with some studies showing efficacy only in the vitamin D‐deficient subgroup. Mesinovic et al. [19] found no significant association between vitamin D metabolites and androgen levels in women with PMOS. These inconsistent results may be related to population characteristics, sample size, and the degree of insulin resistance.

Our study identified a J‐shaped nonlinear association for SHBG and a decline‐then‐plateau pattern for FTI in women, but no robust nonlinear relationships in men. These nonlinear dose–response patterns indicate that the vitamin D–sex hormone relationship is not uniformly linear, and that there may exist a threshold or optimal range of vitamin D status associated with sex hormone homeostasis. Given the cross‐sectional study design, causality cannot be inferred, and the existence of an optimal 25(OH)D range cannot be empirically validated. Accordingly, these nonlinear findings should be interpreted as hypothesis‐generating and do not support immediate clinical recommendations. Prior evidence in men has suggested nonlinear associations between vitamin D and male reproductive endocrine function. Lerchbaum et al. [32] described a U‐shaped relationship between circulating 25(OH)D and hypogonadism risk, with elevated risk at both very low (< 44 nmol/L) and very high (> 102 nmol/L) vitamin D concentrations. Inverted U‐shaped associations with semen parameters have also been reported [33], while other studies have found linear positive correlations between 25(OH)D and sperm quality [34], collectively supporting the concept of an optimal vitamin D range for male reproductive axis function. Gaml‐Sorensen et al. [35] used seasonal variations to estimate vitamin D levels at the onset of spermatogenesis and found that severe deficiency (< 25 nmol/L) was associated with reduced total sperm count and decreased sperm motility. These studies support the possibility of an optimal range for vitamin D’s effects on the male reproductive axis; however, no robust nonlinear relationship was observed in the men in this study, so direct inferences cannot be drawn.

The BMI‐dependent association observed in this study among men differs from the findings of some studies that did not adjust for BMI or included participants with lower BMI. Nimptsch et al. [36] and Wang et al. [37] reported that the positive correlation between vitamin D and testosterone in middle‐aged and older men remained significant after adjusting for BMI, but the mean BMI of the men in those studies was lower (24–26 kg/m^2^), whereas the present study included a larger proportion of obese men, suggesting that the confounding effect of BMI may be more pronounced in obese populations. Rafiq et al. [38] similarly found that the association remained significant after adjusting for BMI in older men, which may be related to the characteristics of sarcopenic obesity in the elderly population. Wehr et al. [7] reported a seasonally consistent positive correlation between vitamin D and testosterone in German men, but that study did not adequately adjust for BMI. With regard to women, Chang et al. [18] reported a positive correlation between vitamin D and total testosterone in healthy, nonobese women (mean BMI of 20.7 kg/m^2^), whereas the present study did not observe a linear positive correlation in the general female population, but only a marginal nonlinear trend. This discrepancy suggests that obesity may modulate the association between vitamin D and testosterone in women.

Strengths of this study include the use of a nationally representative NHANES sample, which enhances generalizability to US adults; rigorous sex‐stratified analyses; application of complex sampling weights; flexible modeling of nonlinear relationships via log transformation and RCS; and sensitivity analyses using bootstrap resampling and exclusion of outliers. However, this study has several limitations. First, as a cross‐sectional study, it can only reveal associations and cannot establish a causal sequence; future prospective cohort or Mendelian randomization studies are needed to validate these findings. Second, sex hormones are influenced by circadian rhythms (morning peak in testosterone) and the menstrual cycle (fluctuations in estradiol), and a single measurement may introduce measurement error; however, since NHANES blood samples were uniformly collected in the morning, this issue was partially mitigated. Third, we were unable to adjust for several potential confounders, including physical activity level, sunlight exposure duration, and dietary vitamin D intake, which may leave residual confounding in fully adjusted models. Fourth, markers of liver health (e.g., alanine aminotransferase, aspartate aminotransferase, fatty liver index, MASLD status) were not included in the analysis. Given that the liver is the primary site of SHBG synthesis and that obesity, vitamin D status, sex hormones, and MASLD are interconnected through multiple pathophysiological pathways, future research should integrate liver health parameters to further elucidate the underlying mechanisms [22].

## 5. Conclusions

In conclusion, this population‐based analysis demonstrates pronounced sex dimorphism in the associations between serum 25(OH)D and circulating sex hormone profiles among US adults. In women, SHBG represents the most robust correlate of vitamin D status: its positive association is independent of BMI and waist circumference, and follows a J‐shaped nonlinear dose–response pattern. In men, by contrast, the associations of 25(OH)D with total testosterone, estradiol, and SHBG are substantially attenuated or rendered nonsignificant after adjustment for adiposity indices, indicating that obesity‐related factors are key confounders of these relationships. Additionally, FTI in women shows a nonlinear decline‐then‐plateau pattern, while estradiol exhibits an approximately linear negative association with 25(OH)D. These findings add population‐based epidemiological evidence to the growing understanding of sex‐specific vitamin D–sex hormone relationships. However, the cross‐sectional design prevents causal conclusions and precludes evidence‐based recommendations for an “optimal” 25(OH)D range. Further well‐powered prospective cohort studies and randomized controlled trials are needed to determine whether vitamin D supplementation can modulate sex hormone levels across sex and BMI subgroups.

## Author Contributions

Yi Han designed the study and wrote the main manuscript text. Data preprocessing and analysis were carried out by Yi Han. Simin Gao reviewed and edited the manuscript.

## Funding

The author(s) received no financial support for the research, authorship, and/or publication of this article.

## Disclosure

All authors have read and approved the final version of this manuscript.

## Ethics Statement

This study was conducted according to the guidelines laid down in the Declaration of Helsinki, and all procedures involving research study participants were approved by the institutional review board of the National Center for Health Statistics (NCHS). Written informed consent was obtained from all participants.

## Consent

The authors have nothing to report.

## Conflicts of Interest

The authors declare no conflicts of interest.

## Supporting Information

Additional supporting information can be found online in the Supporting Information section.

## Supporting information


**Supporting Information 1** Supporting Figure S1 Sensitivity analysis RCS curves excluding the 1st and 99th percentiles of 25(OH)D in men.

**Supporting Information 2** Supporting Figure S2 Sensitivity analysis RCS curves excluding the 1st and 99th percentiles of 25(OH)D in women.

**Supporting Information 3** Supporting Figure S3 Log‐scale scatter plots of 25(OH)D versus sex hormones in men.

**Supporting Information 4** Supporting Figure S4 Log‐scale scatter plots of 25(OH)D versus sex hormones in women.

## Data Availability

The study was based on public databases. The datasets can be found on the NHANES website (https://www.cdc.gov/nchs/nhanes/index.htm).
